# Proteomic and glycosylation biomarkers in rheumatoid arthritis: advancing early diagnosis and precision therapy

**DOI:** 10.1186/s12014-026-09618-z

**Published:** 2026-07-05

**Authors:** Preeti Sharma, Shahbaz Aman, Priya Koundal, Shakeel Ahmed Mohammed, Hardeep Kaur, Faraz Ahmad, Sabiha Khatoon, Shafiul Haque, Arif Hussain

**Affiliations:** 1 Department of Biochemistry, MM Institute of Medical Sciences and Research, Maharishi Markandeshwar (Deemed to be University), Mullana-Ambala, Haryana, 133207 India; 2 Department of Microbiology, MM Institute of Medical Sciences and Research, Maharishi Markandeshwar (Deemed to be University), Mullana-Ambala, Haryana, 133207 India; 3 Present Address: Department of Microbiology, Maharaja Agrasen Kedar Nath Gupta Medical College, Haryana 124507 Bahadurgarh, India; 4 Department of Biosciences & Technology and Central Research Cell, MM Engineering College, Maharishi Markandeshwar (Deemed to be University), Mullana-Ambala, Haryana, 133207 India; 5 Department of Zoology, Ramjas College, University of Delhi, Delhi, 110007 India; 6 Department of Integrative Biology, School of Bio Sciences and Technology (SBST), Vellore Institute of Technology (VIT), Vellore, India; 7 Department of Biochemistry and Physiology, Oklahoma University health Sciences Center, Oklahoma City, OK 73104 USA; 8 Department of Nursing, College of Nursing and Health Sciences, Jazan University, Jazan, Saudi Arabia; 9 School of Medicine, Universidad Espiritu Santo, Samborondon, 091952 Ecuador; 10 Manipal Institute of Health & Life Sciences (MIHLS), Manipal Academy of Higher Education, Dubai Campus, G04, Dubai International Academic City (DIAC), 345050, Dubai, United Arab Emirates

**Keywords:** Biomarkers, Diagnostics, Disease monitoring, Rheumatoid arthritis, Proteomics, Therapeutics

## Abstract

**Background:**

Rheumatoid arthritis (RA) is a chronic autoimmune disease characterized by progressive joint inflammation and damage. Early clinical diagnosis is crucial for effective intervention but presents significant challenges due to an initial asymptomatic inflammatory phase.

**Main body:**

Anti-citrullinated-protein antibodies (ACPA) and rheumatoid factor (RF) detection have been useful in early RA diagnosis, but their reliability is debatable as they are often non-specific. This has prompted a quest for alternative, more robust biomarkers. Researchers have turned to advanced proteomic and glycosylation analyses of biological fluids (e.g., synovial fluid, plasma and serum) and tissues using techniques such as MALDI-MS, Q-TOF, and SELDI-TOF. These approaches may offer a comprehensive approach to scrutinize a range of biomolecules as potential RA biomarkers, from citrullinated-proteins and peptides to novel potential protein biomarkers such as thymosin, macrophage-capping protein, calgranulins, and serum amyloid-A. Further, proteomics-based approaches have the ability to specifically monitor changes in the RA proteome (e.g., glycosylated VCAM1/SEMA4D proteins and GFAP/A1BG auto-antibodies) to unearth potential diagnostic and prognostic candidates. Disease monitoring in response to anti-rheumatic drugs using treatment-responsive markers, such as S100A8/A9 heterocomplex and leucine-rich alpha-2 glycoprotein, is another application of proteomics-based technologies.

**Conclusion:**

In view of the significance of prediction, early diagnosis and monitoring of RA symptomatology, this review discusses the potential utilities of proteinaceous species as proteomics-based biomarkers that may provide insights into disease mechanisms and offer potential avenues for personalized therapeutic interventions to revolutionize RA management.

## Background

**Fig. 1:**
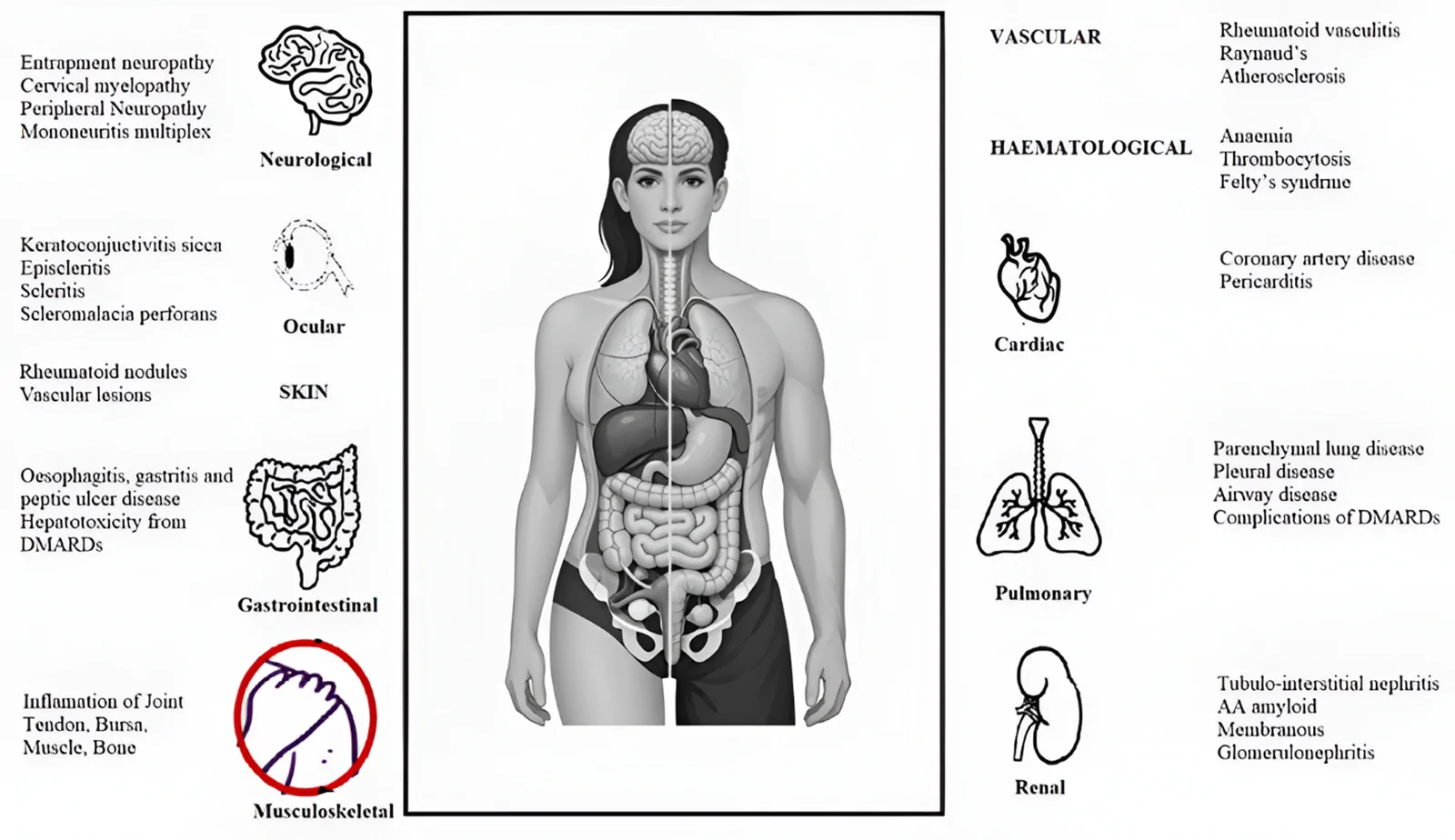
Diagrammatic representation of the vast clinical spectrum of RA that affects major human organs and musculoskeletal joints

**Fig. 2:**
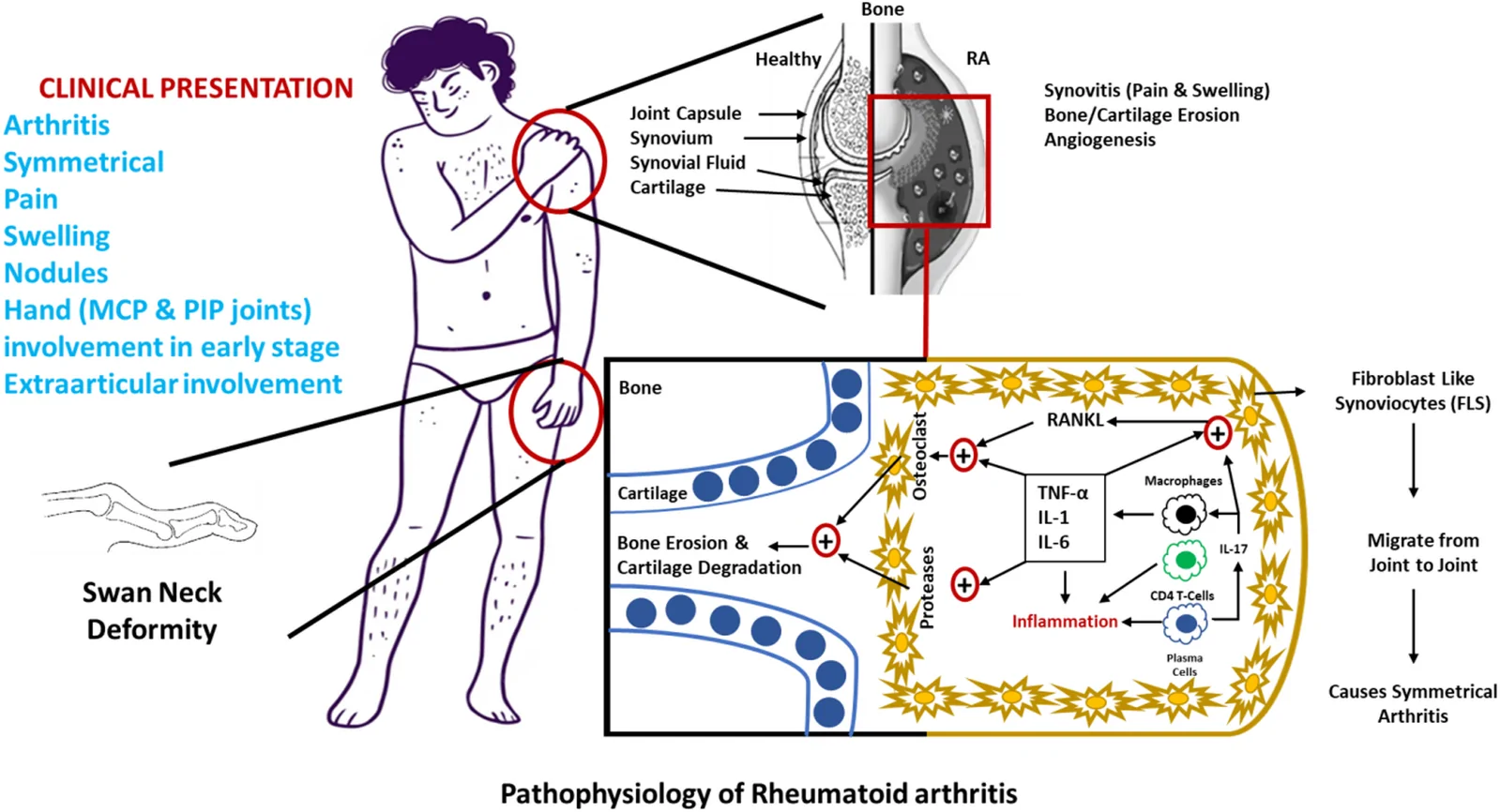
Pictorial demonstration of the pathophysiology of RA including its clinical presentations, structural changes in the joints, and alterations in immune functions

There is a growing global awareness of rheumatoid arthritis (RA), a widespread condition that imposes significant economic and psychological burden on human health [1]. It is estimated that 1% of the global population have RA [2], and it is more common among females and the elderly [3]. While RA is a chronic systemic autoimmune condition primarily affecting the musculoskeletal system, it is often associated with extra-articular manifestations. Indeed, RA presents a broad clinical spectrum, affecting multiple organ systems and musculoskeletal joints, as illustrated in Fig. 1. Nevertheless, joint discomfort and swelling are the most obvious symptoms in RA, which can worsen with time. Tiny joints of the arms and legs are particularly vulnerable, and deteriorations of cartilage and bone can occur as the disease progresses [4]. The pathophysiology of RA, including its clinical manifestations, structural joint changes, and musculoskeletal alterations due to immuno-inflammatory processes, is depicted in Fig. 2.

Early diagnosis and adequate medicine-based therapies are critical for the effective treatment of RA because of the poor prognosis for individuals who do not receive treatment at the disease onset, and the disease’s persistence for decades even after diagnosis. Further, when the condition is severe, patients lose the capacity to function normally and develop permanent impairments [5]. Therefore, monitoring the disease’s progression may help clinicians in selecting the most effective treatment option. Neutrophil infiltration and presence of auto-antibodies, such as anti-citrullinated protein antibodies, in the synovial fluid are pathological hallmarks of RA [6]. Other diagnostic parameters such as morning stiffness, pain, and swelling that persist for at least six weeks are key indicators in patients suspected of having the disease. A full evaluation of RA may include testing for rheumatoid factor (RF), anti-citrullinated protein antibodies (ACPA), C-reactive protein (CRP), and erythrocyte sedimentation rate (ESR), alongside imaging techniques [7]. However, the specificity of ACPA for RA is only up to 75%, and the RF-positive rate among RA patients is about 60%, indicating inadequate diagnostic efficacy. As a result, research aimed at finding reliable biomarkers for RA has gained momentum [8].

In particular, there has been an increase in studies focused on discovering novel diagnostic biomarkers using mass spectrometry (MS). Protein expression and function analyses have been utilized for biomarker identification to determine which mechanisms trigger the changes in protein states and abundances during the onset and progression of diseases such as RA. MS- and proteomics-based methods have the potential to be employed for the serum biomolecular analysis to study protein expression patterns, and to compare the levels of specific biomarker proteinaceous species in healthy and diseased individuals. Furthermore, reports have indicated that the diagnostic biomarkers can be identified in bodily fluids such as synovial fluid, blood and urine [9]. The purpose of this review is to synthesize and delineate recent advancements in the discovery of novel protein-based biomarkers for RA. Beginning with a brief discussion of proteomic biomarker assessment techniques, we focused on specific candidates identified through MS and proteomics, evaluating their potential to improve early diagnosis, predict disease progression, monitor treatment responses, and pave the way for personalized medicinal strategies.

## Utility of proteomics

Proteomics is the study of differential expression profiles of proteins and their various forms present in a particular type of sample which can be used for explaining the altered status of their functional interactions in cellular pathways. Technological advancements in MS and liquid chromatography (LC) tools have tremendously accelerated proteomics studies [10]. Specimens, including body fluids or tissue, must be collected first for the isolation of proteins. It is necessary to use techniques such as affinity depletion, immuno-precipitation, capillary electrophoresis, one- or two-dimensional gel electrophoresis, to isolate the proteins of interest. After the isolation, proteins can be digested by enzymes like trypsin to generate shorter peptide units. Reversed-phase high-performance liquid chromatography (RP-HPLC) can further separate these digested peptides based on their hydrophobicity trait.

In the field of proteome research, MS has shown to be an invaluable tool. MS separates peptides and measures their abundance by ionizing and analyzing them. The three basic components of a mass spectrometer are the ion source, the mass analyzer, and the detector. The mass analyzer determines the m/z ratio of peptide ions produced by the ion source. The m/z range of ions is then tallied by the detector. Matrix-assisted laser desorption/ionization (MALDI) and electrospray ionization (ESI) are two typical techniques for ionizing peptides. There are four primary types of mass analyzers, including ion trap, time of flight (TOF), quadrupole, and Fourier transform ion cyclotron mass spectrometers (FTMS) [11]. With these different types of ion sources and mass analyzers, one can generate results in distinct subfields within MS. Liquid chromatography coupled with electrospray ionization tandem mass spectrometry (LC-ESI-MS/MS) provides a fast, reliable, and simple technique for analyzing proteins. Another commonly used technique is matrix-assisted laser desorption/ionization time-of-flight mass spectrometry (MALDI-TOF MS), or its tandem configuration, MALDI-TOF/TOF MS/MS, when fragmentation-based analysis is required. Collision-induced dissociation (CID) may be used to degrade high-intensity peptides in this tandem MS/MS [12]. De-novo sequencing, peptide mass fingerprinting (PMF), and precise mass and timing tagging can further be employed to identify isolated proteins and peptides by comparing them to databases.

Quantitative proteomics is another set of prominent tools which can be used for protein quantitation employing different methods in specific samples. Labeled methods such as iTRAQ (isobaric tagging for relative and absolute abundances), SILAC (stable isotope labeling of all amino acids in cell culture), and ICPL (isotope coded protein labeling) use tags to label proteins and then compare the labeled samples to determine quantities of specific proteins. Label-free methods such as SWATH (sequential window acquisition of all theoretical fragment ion spectra) are also used for peptide identification as well as quantitation. Instead of differentiating labels, these techniques rely on identifying and comparing the peptides present in different samples to quantify proteins [13]. Researchers can now identify thousands of proteins in a single sample using these techniques. In recent years, quantitative proteomic data has often been integrated with genomic sequences, gene expression patterns, and phenotypic data to create proteome maps and to shed light on biological processes, cell functions, tissue physiology, and bio-fluid (blood, saliva, urine, and tears) profiling. As a prominent example of the gene-protein interface, the Human Proteome Project was initiated to support diverse studies focused on identifying, characterizing, and understanding the proteins produced by human genes [14].

The analysis of human plasma proteome (HPP) may provide a breakthrough in discovering non-invasive and accurate diagnostic, disease monitoring or therapeutic intervention biomarkers. Recent researches on HPP have created a comprehensive map of the plasma proteome, which can be used to identify and validate new diagnostic biomarker candidate molecules. By identifying these biomarkers, it may be possible to develop less invasive diagnostic tests for a variety of diseases [15]. Proteins often work together with other proteins to perform their functions in the body. To better understand these physico-functional interactions, researchers have developed a plethora of MS-based methods. One such method is tandem affinity purification (TAP) tagging, which involves tagging a protein of interest and then isolating it along with any other proteins it physically interacts with. The TAP tag is a heterologous motif that is added to terminus of the protein of interest. It has two components, an IgG-binding calmodulin binding peptide (CBP) and a tobacco etch virus protease (TEV protease) cleavage site. With the help of the TAP tag, the physical complex of which the protein of interest is a part of, can be separated using an IgG matrix. Immuno-precipitation is another method used to separate the protein and its interaction partners. Purification of interacting proteins can then be analyzed using different versions of MS to identify and discern their functional interactions. By studying these protein-protein interactions, we can gain insights into how proteins work cumulatively to perform cellular functions [16]. In conclusion, by using MS-based techniques, it is possible to determine the proteins’ localization, post-translational modifications, and activities, which help in understanding their pathophysiological relevances [17].

### Proteomics approach for identifying novel biomarkers for RA

An initial phase characterized by under-expressed clinical symptoms and low-grade inflammation often leads to underdiagnosis of RA [18]. Nevertheless, studies indicate that immunological reactions that drive RA pathophysiology may initiate at extra-articular sites rather than within the joints themselves [19]. Progressive aggravations in the immune function dysfunction may allow spread of the outcomes to musculoskeletal sites, and even to locations where the immune system has direct interaction with environmental components, such as the mucosal surface in the lungs and stomach [20]. Proteomic evaluation of immune system components may therefore be used for predictive and diagnostic purposes in RA.

There are two major forms of auto-antibodies that can be detected in a patient’s blood, viz. RF and ACPAs. In fact, ACPAs can be detected in the patients’ blood much earlier before the appearance of overt symptoms of RA, emphasizing their potential in early prodromal diagnosis of the condition. While ACPAs are more strongly linked to the development of RA, they can also be detected in cases with rheumatic fever [21]. In this regard, ACPAs against cyclic citrullinated peptides may serve as better early diagnostic indicators for RA [22]. Although detection of ACPAs is often used for early diagnosis of RA, research indicates that only 60% of subjects with positive tests for ACPAs may develop RA later on, raising questions on the efficacy and sensitivity of ACPAs as specific early diagnostic markers for RA [23]. Correspondingly, individuals at a high risk for RA may not have detectable amounts of ACPA antibodies or RF, which further hinders prediction and early diagnosis of RA. Therefore, it is imperative to discover new proteinaceous and auto-antibody-based markers for RA subjects, especially those who lack RF or ACPA antibodies [23]. Indeed, in recent years, there has been a surge in identifying more sensitive and specific biomarkers with potential utilities in early RA diagnosis and/or in predicting onset of RA symptomatology [24].

#### Biological fluids

Researchers have been exploring proteomic changes in biological fluid samples of RA cases in order to discern thepathophysiological mechanisms and identify novel biomarkers. One of the candidates with potential diagnostic value are the citrullinated proteins [25]. Citrullinated proteins in RA synovial tissue and other biological fluids have been identified and validated using two-dimensional gel electrophoresis coupled with mass spectrometry (2-DE-MS) and Western blotting [26]. MALDI-MS analysis has also been used to compare protein distribution in synovial tissue samples from subjects with RA and osteoarthritis (OA), discerning potential differentiating markers [27]. Synovial fluid samples have also been envisioned as sources of identifying new biomarkers for RA using label-free MS-based methods [28]. Interestingly, macrophage-capping protein (CAPG) was found to be more prevalent in synovial fluids of RA compared to OA cases, as indicated by iTRAQ and multiple reaction monitoring (MRM) techniques [29]. Chaperone GRP78/BiP is a protein found in human saliva, and evidently has higher expression in case of RA patients, compared to the saliva of healthy individuals, indicating its possible use as a salivary marker [30]. Surface-enhanced laser desorption/ionization-time of flight (SELDI-TOF) technology has been used to find possible biomarkers for RA in serum, including some calgranulins and new forms of serum amyloid-A protein [31]. A recent study found that calgranulins proteins (S100A8, S100A9 and S100A12) may be used for differentiating RA from other joint diseases [32]. Amounts of these proteins in sera and synovial fluids may also serve as early diagnostic marker of RA [33]. Similarly, serpin A11, complement factor H-related protein-5, cartilage acidic protein-1, and coagulation factor-IX have been proposed as differentiating entities between RA and psoriatic arthritis [34]. Researchers have also found post-translationally modified proteins in synovial fluid as potential diagnostic agents. For instance, RA subjects have certain peptides with fraction of citrulline and phosphorylated serine residues in their synovial fluid which may be unique to RA, and not found in other inflammatory joint diseases [35]. Indeed, citrulline-specific enrichment strategy coupled with LC-MS/MS may be used to study “citrullinome” changes in RA synovial fluid [36]. Another study examined synovial fluids from RA patients using 2D strong cation exchange-reversed phase (SCX-RP) LC-MS/MS to identify several citrullinated proteins, including fibrinogens and immunoglobulins [37].

Another post-translational modification, glycosylation, has been investigated as a potential body fluid-based biomarker in RA, with aberrant IgG Fc glycosylation emerging as one of the most reproducible molecular signatures of the condition. Thus, IgG antibodies, particularly ACPAs, derived from RA patients exhibit significantly reduced Fc-linked galactosylation and sialylation compared to healthy individuals [38]. Using LC-ESI-MS–based glycoprofiling, the authors showed an increased proportion of agalactosylated Fc glycoforms (G0F) alongside marked reductions in terminally sialylated forms (G1FS1, G2FS1, and G2FS2) of IgG in RA. Notably, this hypogalactosylated and hyposialylated Fc glycosylation pattern was observed consistently in both total serum IgG and ACPA fractions from RA patients, and correlated with enhanced inflammatory potential of the antibodies. Functionally, reduced Fc sialylation was shown to augment arthritogenic activity, whereas enforced sialylation of ACPAs attenuated joint inflammation in collagen-induced arthritis models, highlighting the direct immunomodulatory role of IgG glycosylation in RA pathogenesis. These findings strongly support glycan modifications, particularly Fc galactosylation and sialylation status of IgG as potential clinically relevant biomarkers with diagnostic, prognostic, and therapeutic implications in RA.

Multiple investigations have used lecithin affinity chromatography to identify novel glycoproteins in the plasma of RA patients, which were then analyzed by mass spectrometry [39]. Recent studies have found new glycosylated proteins that can be used as biomarkers for RA in synovial fluid [40]. Additionally, proteomic studies have identified several proteins that are expressed differently in the blood of people with RA and OA, which could be used as a protein signature for these diseases [41, 42]. Identification of early diagnostic biomarkers for RA, have found two proteins (STEAP4 and ZNF 648) that might help to diagnose the disease in its early stages [35].

Proteomics is used to find new ways to diagnose and treat RA. Researchers have looked at synovial fluid and serum to find proteins that could serve as biomarkers for the disease. For instance, in a study, researchers found that glycosylated proteins, such as VCAM1 and SEMA4D, could be used as biomarkers for RA [35]. Additionally, proteomics has been used to look at auto-antibodies and found that auto-antibodies to GFAP and A1BG could be used as diagnostic tools for RA. Recent studies have used antigen bead arrays to screen for autoimmunity status in RA serum samples. Overall, proteomics has the potential to identify new diagnostic tools and treatment options for RA [43]. The use of antibody and antigen arrays have been further shown to be multiplex, automated, and repeatable, and capable of specifically identifying RA patients whether they are sero-positive or sero-negative.

### RA-related tissues

The significance of biomarkers in diagnosing RA cannot be overstated, as they serve as powerful tools for identifying the disease early and initiating timely interventions, ultimately leading to improved patient outcomes. These biomarkers can significantly affect treatment response, disease progression, and quality of life in RA patients. RF, anti-CCP antibodies, CRP, ESR, and synovial fluid analysis are only some of the biomarkers used to better understand the disease and personalise treatment. A variety of studies have indicated the relevance of RA-related tissues as sources of biomarkers. For instance, a study indicated that early-stage RA patients’ bronchial tissue and synovial tissue shared two citrullinated vimentin peptides, identifying lung as a potential origin site for the autoimmune responses associated with the condition [44]. Early onset of RA has also been linked to alterations in the gut flora [If space is missing before "[44]", please put it, as we are unable to change this.^45^]. Recently, the proteome of the colon mucosa of RA patients and healthy controls was examined [46]. Three citrullinated peptides were only detected in RA biopsies, and there was a significant increase in dihydrofolate reductase (DHFR) in RA biopsy samples, compared to controls. This confirms that ACPA, an early marker of RA development, is produced in connection with mucosal surfaces [35]. The ability to diagnose RA in its nascent stages using RA-associated tissue/biopsy samples may empower clinicians to intervene early, mitigating joint damage, and curbing the relentless progression of the disease. Furthermore, these biomarkers provide an invaluable means of monitoring disease activity, allowing for the assessment of treatment efficacy and the adjustment of therapeutic interventions to achieve optimal patient outcomes. Additionally, certain biomarkers can act as prognostic indicators, affording clinicians the ability to predict disease trajectory and anticipate potential complications [47]. This prognostic insight enables the development of personalized treatment plans, catering to each patient’s unique needs, thereby maximizing therapeutic outcomes. The impact of biomarkers extends beyond the clinical realm, as their utilization positively influences patient-reported outcomes, including pain reduction, improved physical function, and enhanced overall well-being. By harnessing the power of biomarkers, healthcare providers can deliver a more personalized approach to RA management, revolutionizing the field and bringing hope to those burdened by this debilitating condition [48]. As the realm of biomarker research continues to expand, we stand at the precipice of an era characterized by even greater advancements in RA diagnosis, treatment, and patient care, underscoring the profound impact of these biomarkers on the lives of subjects affected by this chronic condition [49].

### Emerging biomarkers for diagnosis of RA

RF and antibodies to citrullinated peptides are the most important clinically effective biologic markers for the diagnosis of RA. There is a correlation between the presence of RF or ACPA and worsening functional and radiographic results. However, neither test is specific enough to diagnose RA on its own, and prognosis differs considerably between sero-positive and sero-negative patient populations [50]. To put it simply, RF are auto-antibodies that specifically attach with the Fc region of IgGs. Interstitial lung disease and vasculitis are extraarticular symptoms associated with a more severe disease course when RF levels are high. A good response to rituximab following the failure of tumor necrosis factor (TNF) inhibitor therapy is also more likely when RF is present. Since ACPA are relatively specific for RA, they can be used in the differential diagnosis of early polyarthritis. A frequently used ACPA assay detects antibodies against mutant citrullinated vimentin, and ELISAs for antibodies against CCP are also widely employed. Patients who are properly treated, especially those on disease-modifying antirheumatic drugs (DMARDs) therapy in the early stages, often see a reduction in their ACPA titter [51]. Anti-CCP assays have emerged as the most common ACPA detection tools in the market. Second-generation anti-CCP antibody tests (anti-CCP2) are an example of an updated assay protocol with increased sensitivity and specificity, compared to the first-generation anti-CCP assays. Autoimmune rheumatic disorders, TB, and chronic pulmonary diseases are only a few examples of conditions in which a positive ACPA test is seen [52]. Based on data from patients, ACPA testing may be more reliable than RF for predicting erosive disease. The radiographic progression of RA is more rapid in patients who test positive for ACPA in the early stages of the disease. Radiographic progression also appears to be more likely in IgM-RF-negative patients with early oligo- or polyarthritis who test positive for ACPA. Radiographic damage progression may be predicted in 83% of patients with anti-CCP testing for ACPA. Antibodies against other citrullinated proteins have been detected in RA patients. Fibrinogen, vimentin, synthetic type I and type II collagen telopeptides, alpha-enolase, and other proteins can be citrullinated. Table 1 details the most potential biomarker for RA [53].

**Table 1:** Potential biomarkers for diagnosis of RA

Protein	Pathophysiological implications in RA	Ref.
IL-13	Diagnostic or prognostic tool for RA; valuable for early detection and disease progression	[54]
14-3-3η protein	Contributes to inflammation promotion, cartilage degradation, and bone erosion.	[55]
Adiponectin	Supports joints deterioration and bone degradation in RA	[56]
Anti-citrullinated protein antibodies (ACPA)	Targets citrullinated proteins, leads to immune complexes formation and chronic inflammation in joints	[21]
Anti-peptidyl-arginine deaminase 3 (PAD3)	Anti-PAD3 antibodies promote inflammation, immune responses, and joint damage	[57]
Carboxypeptidase A1 (CPA1)	Indirectly linked to RA, but with unknown functions	[58]
C–C motif chemokine 11 (CCL11)	Overproduced in synovial fluid and affects RA patient’s tissue	[59]
C–C motif chemokine-22 and 17 (CCL22 and CCL17)	Inflamed joints produce CCL22 and have regulatory T cells which modulate immune response to increase immune tolerance; CCL17 may maintain chronic inflammation in RA via memory T cells recruitment; synovial fluid and damaged tissue have increased levels of CCL22 and CCL17	[60]
Cluster of differentiation 20 (CD20)	Highly B cells specific; therapeutic antibodies (rituximab) target CD20 in RA and deplete B cells as well as modulate immune response	[61]
Clusterin	Usually present in high levels in patients of early RA	[62]
Leptin	Adipose tissue hormone leptin involves prolonged inflammation and joint degeneration in RA	[63]
Lipoprotein A (Lp-A)	Causes joint injury by raising inflammatory responses and impairing blood vessel function	[64]
Mucin-1 subunit alpha (MUC1-α)	Contributes to the development and progression of RA via immune cell activation and inflammatory cytokines release	[65]
Neprilysin (NEP)	Dysregulation causes joint injury and increased inflammation, and regulates inflammation and tissue remodelling in RA	[66]
Orosomucoid (ORM 1, ORM2)	Upregulated in RA and linked with disease pathogenesis	[67]
Presepsin and procalcitonin	May increase in RA, suggesting the presence of an infection or a more severe inflammatory response	[68]
Signal peptidase complex catalytic subunit (SPCS)	Increased in RA and may have a role in the pathophysiology of the disease by aiding in the generation of pro-inflammatory cytokines and chemokines	[69]
Macrophage-capping protein (CAPG)	Overexpressed in RA synovial fluid compared to osteoarthritis; involved in actin cytoskeleton remodeling, macrophage migration, and synovial inflammation; Proposed as a potential diagnostic biomarker for RA	[30]

## Biomarkers for monitoring disease state and severity

Assessing RA patients is primarily based on disease status levels, which are associated with progressive joint degeneration that characterizes the disease. Joint discomfort, edema, and an elevated ESR or CRP protein level are all components of the Disease Activity Score (DAS28), the most widely used metric for assessing RA severity. However, this approach has drawbacks such as requiring extra time, expertise, and not providing sufficient information to patients for informed decision making [70]. Radiology is often used to assess disease severity, but indicators of future incapacity, such as radiographic evidence of structural damage, may not become apparent until one to three years after the initial signs of the disease have appeared. Furthermore, there are currently no early biomarkers capable of predicting a high risk of joint damage and dysfunction [71]. In one study label-free LCMS/MS analysis was performed to quantify urinary proteome profiling on urine samples from individuals with RA and OA [72]. This profiling allowed the authors to distinguish 134 DEPs in urine samples from people with RA and OA. They found that urine soluble CD14 (sCD14) had diagnostic value comparable to that of conventional blood measurements by evaluating the 134 differentially expressed proteins (DEPs) alongside the alterations in the mRNA expression profiles. The predictive power of their model increased further when serum CRP was included [67]. Serum levels of CRP, S100A8 (calgranulin A), S100A9 (calgranulin B), and S100A12 (calgranulin C) were found to be higher in patients with erosive disease compared to those with nonerosive RA or healthy people in another investigation [73]. Semi-quantitative protein profiling of diseased tissues was used to identify possible biomarkers, and then validated using quantitative multiple reaction monitoring (MRM) analysis of peripheral blood. Through these methods, authors were able to identify about 33 potential RA biomarker candidates, all of which had the potential to serve as indicators of disease severity. Biomarkers have also been sought by other research groups using two-dimensional liquid chromatography in tandem with mass spectrometry [9]. proteomic processes could be made more effective with the use of improved sample enrichment and separation technologies, as well as more precise mass spectrometers. Major proteomics markers and their involvement in tracking the severity of RA are summarized in Table 2.

**Table 2:** Potential biomarkers for monitoring RA progression and severity

Diagnostic biomarker	Pathophysiological implications in RA	Ref.
Alpha-1-acid glycoprotein 2 (AGP2)	Regulates immune response modulation, inflammation regulation, and cytokine generation	[74]
Anti-CarP antibodies	Promote inflammation and immunological dysregulation leading to development of RA	[75]
Cartilage oligomeric matrix protein (COMP)	Elevated level of COMP in synovial fluid and serum leads to joint inflammation and structural damage	[76]
C–C motif chemokine 5 (CCL5)	Elevated level of CCL5 stimulates T cells and monocytes causing inflammation of joints	[77]
Collagen alpha-1(I) chain	Primarily works to maintain structural integrity of joints and contributes in collagen fibrils synthesis; but in RA, it modifies and causes breakdown of cartilage and bone erosion	[78]
Collagen alpha-1(III) chain	Dysregulation enhances immune cell activation and cytokine release, which cause chronic inflammation and destruction of joints	[79]
Collagen alpha-1(VIII) chain	Elevated level signifies its involvement in angiogenesis, inflammation, and tissue remodeling in RA	[80]
Collagen alpha-1(XV) chain	Dysregulation alters tissue remodeling and causes inflammation and structural abnormalities	[80]
Collagen alpha-2(I) chain	Dysregulation damages cartilage and bone and have possible linkage in the development and progression of RA	[35]
C-reactive protein (CRP)	An inflammatory marker estimation of which may provide an insight into disease stage and therapy progress	[81]
Serum amyloid A4 and vitamin D binding protein	Plays a role in mediating immunological dysregulation and joint inflammation which is associated with RA	[9]
Transthyretin	Has anti-inflammatory activities and acts as defense mechanism against RA; the severity of RA has been linked to lower levels	[82]

## Biomarkers for assessing treatment response

RA is a disease that can progress in different ways and responds differentially to different treatments. Its course can be mild, moderate, or severe and gets worse over time. Therefore, biomarkers for disease prognosis could aid in the identification of patients who are more likely to worsen over time so that the most aggressive treatments may be provided to them [48]. One way to ensure that anti-RA treatment is effective and to predict adverse reactions before they become permanent is to monitor drug treatment closely. However, some patients may not respond well to treatment throughout the course of their illness. As a result, it is necessary to closely monitor patients at each clinical visit to adjust treatment doses based on treatment effectiveness [83]. In recent years, several medicinces have been proposed for the management of RA. These include IL-1 antagonists like anakinra, IL-6 antagonists like tocilizumab, T cell co-stimulation blockers like abatacept, and B cell depletors like rituximab. However, due to the wide range of responses among patients, many of these either do not improve disease outcomes with the treatment, or have major adverse effects [84].

Proteomic studies have been conducted in order to monitor changes in RA patients upon provision of therapeutic regimens, such as anti-TNF-based paradigms [85]. For instance, two-dimensional analysis was utilized on samples of synovial fluid and plasma from RA patients in an early investigation. Measuring the S100A8/A9 heterocomplex in blood may be a valuable biomarker for tracking the efficacy of etanercept treatment [86, 89]. Later LC-MS/MS analysis of plasma from RA patients using etanercept found that 12 proteins were downregulated and 5 were upregulated. Some of these proteins have been linked to RA and could be used as therapeutic outcome markers [87]. Quantitative proteomics using iTRAQ has also been utilised to track the effects of anti-TNF treatment. Several TNF-alpha-regulated blood proteins were found to be downregulated in patients who responded well to infliximab in one investigation [88].

Studies using iTRAQ-based quantitative proteomics have also suggested that leucine-rich alpha-2 glycoprotein (LRG) levels could be used as a biomarker to track the effectiveness of TNF blockers, with two of them showing that LRG levels correlate with disease activity in patients treated with infliximab or etanercept [90, 91]. Other authors have independently confirmed the potential of LRG as a marker of RA disease activity [92]. More recently, a study using SOMAScanTM affinity proteomics identified serum IL-16 as a possible indicator of how early treatment with adalimumab and tocilizumab affects RA, while protein arrays were used to find that a small group of 12 cytokines could be used to track rituximab treatment in patients with long-standing and active RA [93]. A recent study utilized multiplex bead array technology to measure the changes in levels of IgA and IgG against citrullinated antigens in RA patients who were on rituximab medication. The levels of IgG and IgA to 30 different citrullinated antigens varied between patients and changed during therapy [94].

Disease-modifying anti-rheumatic drugs (DMARDs) used to treat RA, include traditional DMARDs, such as methotrexate, hydroxychloroquine, sulfasalazine, and leflunomide, as well as targeted synthetic DMARDs, such as tofacitinib, which is designed to target janus kinases (JAKs) [95]. The advent of DMARDs and other biologics, has revolutionized the treatment of RA in recent years. Some of these include TNF inhibitors, such as abatacept and T cell co-stimulation inhibitors, rituximab and anti-B cell-targeting therapeutics, as well as tocilizumab and other monoclonal antibodies that block IL-6R [96]. Despite the widespread availability and usage of these therapeutics, information of their effectiveness is largely limited. This has raised the need for biomarker-based research to assess drug efficacy. For instance, TNF inhibitors are the most commonly used type of biological treatment for RA, but they are not effective for approximately one-third of patients, and they are more expensive than traditional DMARDs. Using biomarkers to evaluate the effectiveness of anti-TNF therapy could lead to better disease management and reduce healthcare costs.

As indicated, recent research has considerably shifted focus on the identification of new biomarkers for assessing managment of RA cases. In this regard, 2D LC-MS/MS analysis to investigate proteins that were expressed differently in the serum or plasma after anti-TNF treatment has identified key protiens, FAM62A/MBC2 and CTGF, involved in the metabolism of articular cartilage and the activation of NF-kappa B, as potential candidates [97]. Further, whole-blood transcriptome profiling is being investigated for the identification of molecular markers that can predict how RA patients will respond to rituximab [98]. The results suggest that downregulation of the interferon pathway and upregulation of inflammatory pathway proteins, including NF-kB, IL33, and STAT5A, were the main features of the European Alliance of Associations for Rheumatology (EULAR) responder group’s protein signature. Therefore, it is likely that novel biomarkers that can predict responders and non-reponders in RA management will be developed in the near future. These biomarkers may also help identify patients who are most likely to have serious side effects during the therapy. Additionally, since anti-RA therapies are generally expensive and their effectiveness varies amongst patients, finding biomarkers to predict how well a treatment will work, will aid in the management of the condition and improve cost-effectiveness [99]. Table 3 represents the potential proteomic biomarker for evaluating therapeutic interventions in RA, while Table 4 elaborates the implications of the major proteomicbased biomarkers and biotargets identified for diagnostic and prognostic purposes.

**Table 3:** Potential biomarkers for assessing therapeutic intervention in RA

Diagnostic biomarker	Pathophysiological implications in RA	Ref.
Alpha-1-microglobulin (A1M)	Because of its antioxidant and anti-inflammatory qualities, A1M may reduce joint inflammation and damage	[100]
Alpha-2-HS-glycoprotein (AHSG)	In RA, AHSG has a role in controlling both calcification and inflammation	[101]
Alpha-2-macroglobulin (A2M)	A2M behaves as a molecular sponge by reducing inflammation and protecting joint tissue	[102]
Apolipoprotein A2	ApoA2 may contribute to inflammation and joint damage in RA	[103]
Beta-2-glycoprotein 1	Increased level of β-2GP1 detected in RA may be associated with inflammation and joint damage	[104]
Cluster of differentiation 20 (CD20)	Dysregulation of CD20 alters tissue remodeling and causes inflammation and structural abnormalities	[61]
C-X-C motif chemokine 13 (CXCL13)	CXCL13 recruits and functionally regulates B and T cells within the synovium and promotes the creation of ectopic lymphoid structures; elevated levels of CXCL13 have been associated with severity of RA	[105]
C-X-C motif chemokine 16	It activates immune cells including T cells and macrophages in the inflammed synovia; When CXCL16 levels are high, joint inflammation is enhanced in	[106]
Fibulin-3	Damage to joints caused during RA has been linked to fibulin-3’s role in the remodeling and preservation of connective tissues	[107]
Heme oxygenase-1 (HO-1)	Reduces inflammation and prevents oxidative damage, thereby regulating RA pathogenic mechanisms	[108]
Histidine-rich glycoprotein	In RA, HO-1’s anti-inflammatory and antioxidative characteristics aid in immune response regulation and protection against oxidative damage	[109]
Inter-alpha-trypsin inhibitor heavy chain H1	ITIH1 is involved in the regulation of inflammation and tissue remodeling in RA	[110]
Inter-alpha-trypsin inhibitor heavy chain H2	ITIH2 is an acute-phase protein that modulates the immune responses and extracellular matrix dynamics in RA	[110]
Interleukin IL-9 (IL-9), TNF-α, vascular endothelial growth factor (VEGF)	IL-9, TNF-α, and VEGF are all involved in the pathogenesis of RA, leading to synovial inflammation and pannus formation	[111]
Kallikrein 2	Inhibiting kallikrein 2 showed promising therapeutic efficacy in RA	[112]
Kallikrein 4	Joint inflammation and tissue damage are caused by kallikrein 4, an enzyme implicated in the destruction of extracellular matrix in RA	[112]
Kallikrein 5	Kallikrein 5 contributes to inflammation and tissue damage in RA, likely by activating pro-inflammatory cytokines and degrading extracellular matrix components	[112]
Kallikrein 6	Kallikrein 6 increases inflammation and tissue damage in RA, likely by activating pro-inflammatory mediators and destroying extracellular matrix components	[112]
Kallistatin	Kallistatin has shown potential as a therapeutic target in RA by attenuating joint inflammation and angiogenesis	[112]
Leptin	Leptin plays a role in RA-associated prolonged inflammation and joint degeneration	[113]
Plasminogen	Activation of plasminogen contributes to joint destruction and inflammation in RA	[114]
Protein S100-A9	It recruits and activates immune cells and induces the production of pro-inflammatory cytokines, ultimately leads to tissue destruction	[115]

**Table 4:** Biomarkers and biotargets with therapeutic and diagnostic potential against RA

Diagnostic biomarker	Pathophysiological implications	Ref.
Anti-citrullinated protein antibodies (ACPA)	ACPA targets citrullinated proteins, leading to the formation of immune complexes and chronic inflammation in the joints	[21]
Anti-peptidyl-arginine deaminase 3 (PAD3)	Anti-PAD antibodies promote inflammation, immune responses, and joint damage	[57]
Bone morphogenetic protein (BMP)	BMP-1 is primarily involved in regulating bone formation and remodeling; its dysregulation leads to degradation of collagen, potentially influencing joint tissue integrity and inflammation in RA	[116]
Calpain 2	High levels of calpain 2 are associated with heightened imflmmation and tissue damage in RA	[117]
Calprotectin	Calprotectin is produced by active immune cells and contributes to local inflammation and joint destruction	[118]
Cathepsin S	Cathepsin S is overproduced and overactive in RA, and causes inflammation and joint damage by degrading extracellular matrix components	[119]
C-X-C motif chemokine 10 (CXCL10) and C-X-C motif chemokine 13 (CXCL13)	CXCL10 and CXCL13 levels are linked with persistent inflammation and immune cell infiltration in RA	[120]
Gelsolin	Gelsolin is an actin-binding protein, and controls synovial hyperplasia and joint degeneration	[121]
Granzyme A	Granzyme A induces apoptosis leading to joint destruction	[122]
Interleukin 6 (IL-6)	Inhibitors of IL-6 signaling have been found to be clinically useful in lowering inflammation and improving disease outcomes in RA patient	[123]
Interleukin 6 receptor (IL6R)	Targeting IL6R has been shown to reduce inflammation and improve outcomes in RA patients	[124]
Interleukin-13 (IL-13)	IL-13 is an important cytokine in RA, and is associated with chronic inflammation and joint destruction	[54]
Interleukin-17 (IL-17)	IL-17 is a pro-inflammatory cytokine that promotes chronic inflammation and joint damage in RA	[125]
Intra-cellular adhesion molecule-1 (ICAM-1)	ICAM-1 plays a key role in RA by facilitating the adhesion and migration of immune cells	[126]
Matrix metalloproteinase-1 (MMP-1)	MMP-1 degrades collagen, leading to joint inflammation, synovial hyperplasia, and progressive joint damage in RA	[127]
Matrix metalloproteinase-12 (MMP-12)	MMP-12 contributes to joint damage in RA by degrading extracellular matrix components	[128]
Matrix metalloproteinase-13 (MMP-13)	MMP-13 is a key player in RA, contributing to cartilage destruction and joint damage, making it a potential therapeutic target	[129]
Matrix metalloproteinase-14 (MMP-14)	MMP-14 is involved in the pathogenesis of RA by degrading extracellular matrix components and promoting inflammation	[130]
Matrix metalloproteinase-7 (MMP-7)	MMP-7 degrades extracellular matrix components and activates other matrix metalloproteinases	[131]
Matrix metalloproteinase-9 (MMP-9)	In RA, MMP9 causes inflammation and tissue damage by degrading extracellular matrix components	[132]
Orosomucoid 1 (ORM-1)	α1-Acid glycoprotein (AGP) or orosomucoid-1 modulates the immune system, potentially affecting RA progression	[133]
Osteoprotegerin (OPG)	The disruption of the equilibrium between OPG and receptor activator of nuclear factor kappa-B ligand (RANKL) in RA contributes to the dysregulation of bone homeostasis	[133]
Resistin	Resistin is a cytokine secreted by adipocytes and immune cells that promotes inflammation in RA patients	[134]
Retinol-binding protein 4 (RBP-4)	RBP-4 is an adipokine secreted by adipocytes that plays a role in insulin resistance and metabolic dysfunction in RA	[135]
Serum Amyloid A1 (SAA1)	SAA1 contributes in pathogenesis of RA by activating immune cells, enhancing pro-inflammatory cytokine release, and promoting amyloid deposits	[136]
Tumour necrosis factor α (TNF-α)	TNF-α drives inflammation and joint degeneration; targeting TNF-α has been shown to be beneficial at controlling RA symptoms	[137]
Vascular endothelial growth factor (VEGF)	Targeting VEGF to inhibit angiogenesis may reduce disease severity in RA	[138]

## Limitations of proteomic techniques

We culminate our study with a brief discussion of the limitations of the proteomic techniques for RA research and management. The main focus is on liquid LC-MS, one of the proteomic approaches which has emerged as a promising tool for identifying novel biomarkers for RA. The limitations of this technique are summarized below.

1. Sample Complexity: One of the primary limitations of LC-MS-based proteomics is the complexity of biological samples. In RA, the synovial fluid and tissue samples are complex mixtures of proteins that vary in concentration and post-translational modifications. The presence of high-abundance proteins such as albumin and immunoglobulins can also interfere with the detection of low-abundance proteins. Theefore, the complexity of the sample can lead to poor reproducibility and sensitivity in identifying novel biomarkers.
2. Dynamic Range: The dynamic range of protein concentration in biological samples is large, with some proteins being present at high concentrations while others are present at low concentrations. This can make it difficult to detect low-abundance proteins in the presence of high-abundance proteins. The use of different sample preparation methods, such as depletion or enrichment of certain protein classes, can help to overcome this limitation.
3. Quantification: Quantification of protein expression is another limitation of LC-MS-based proteomics. Quantification is often performed using label-free or stable isotope labeling methods, but both methods have limitations. Label-free methods have limited accuracy, while stable isotope labeling methods can be expensive and complex to perform.
4. Data Analysis: The large amount of data generated by LC-MS-based proteomics can be challenging to analyze. The use of bioinformatics tools is essential for the identification of potential biomarkers, but the identification of true biomarkers requires careful validation and confirmation in independent cohorts.
5. Standardization: Standardization of LC-MS-based proteomics is essential for the identification of reliable biomarkers. Lack of standardization in sample preparation, LC-MS instrumentation, and data analysis can lead to poor reproducibility and low sensitivity in identifying novel biomarkers.

In summary, LC-MS-based proteomics is a powerful tool for identifying novel biomarkers for RA, but it has several limitations that need to be addressed. The complexity of the sample, dynamic range, quantification, data analysis, and standardization are important factors that need to be carefully considered when using this approach.

## Conclusions

The field of proteomics has played a pivotal role in advancing our understanding of RA by identifying and characterizing potential biomarkers associated with the disease. The conventional diagnosis of RA relies heavily on clinical manifestations, often leading to delayed recognition due to an initial period of inflammation without apparent symptoms. Autoantibodies, particularly RF and ACPAs, have been crucial in early detection, with ACPAs showing promise as potential indicators even before the onset of symptoms. However, the reliance on RF and ACPA antibodies has its limitations, as not all individuals at high risk for RA exhibit these markers. Contradictory findings regarding the effectiveness of ACPAs in predicting the development of RA have prompted a quest for alternative biomarkers with greater sensitivity and specificity. Proteomics approaches have delved into the analysis of biological fluids, RA-related tissues, and novel citrullinated proteins to uncover potential candidates for early diagnosis and prognosis. Studies exploring citrullinated proteins in RA synovial tissue and biological fluids have identified various candidates, including thymosin, CAPG, and chaperone GRP78/BiP. Advances in MS techniques have facilitated the discovery of novel citrullinated proteins and post-translational modifications, shedding light on the molecular landscape of RA. Glycosylation patterns, especially in the synovial fluid, have emerged as potential biomarkers, highlighting the diversity of proteomic investigations in RA. The exploration of RA-related tissues has revealed connections between bronchial tissue alterations and synovial tissue in early RA patients, suggesting lung as a potential origin point for the autoimmune response. Mucosal surfaces, including the colon mucosa, have been implicated in the production of ACPAs, further emphasizing the systemic nature of RA pathology. The identification of citrullinated peptides specific to RA biopsies underscores the importance of understanding the disease at its molecular level. Biomarkers not only aid in early diagnosis but also play a crucial role in monitoring disease activity and severity. Traditional markers such as CRP and ESR, along with newer proteomic markers like soluble CD14 (sCD14) and S100 proteins, contribute to the assessment of disease severity. The advent of DMARDs has revolutionized RA treatment, and proteomic studies have strived to identify biomarkers for assessing treatment responses. Proteomic analyses have revealed potential markers for monitoring the efficacy of anti-TNF therapy, offering insights into personalized treatment strategies. In the pursuit of precision medicine for RA, the identification of biomarkers extends beyond diagnosis and disease monitoring to predicting treatment responses. The potential of proteomics to unravel the intricacies of RA pathogenesis and therapeutic interventions underscores its significance in advancing patient care. As research in this field continues to evolve, the identification of reliable proteomic biomarkers holds the promise of transforming the diagnosis, prognosis, and treatment of RA, ultimately improving outcomes and quality of life in individuals affected by this complex autoimmune disorder.

## Data Availability

No datasets were generated or analysed during the current study.

## Abbreviations

ACPA: Anti-Citrullinated Protein Antibody
A1BG: Alpha-1B-Glycoprotein
CAPG: Macrophage-Capping Protein
CBP: Calmodulin Binding Peptide
CCP: Cyclic Citrullinated Peptide
CID: Collision-Induced Dissociation
CRP: C-Reactive Protein
CTGF: Connective Tissue Growth Factor
DAS28: Disease Activity Score in 28 Joints
DEP: Differentially Expressed Protein
DHFR: Dihydrofolate Reductase
DMARD: Disease-Modifying Anti-Rheumatic Drug
ELISA: Enzyme-Linked Immunosorbent Assay
ESI: Electrospray Ionisation
ESR: Erythrocyte Sedimentation Rate
FTMS: Fourier Transform Ion Cyclotron Mass Spectrometry
GFAP: Glial Fibrillary Acidic Protein
GRP78/BiP: Glucose-Regulated Protein 78/Binding Immunoglobulin Protein
HPP: Human Plasma Proteome
ICPL: Isotope-Coded Protein Labelling
IgG: Immunoglobulin G
IL: Interleukin
IL-6R: Interleukin-6 Receptor
iTRAQ: Isobaric Tagging for Relative and Absolute Quantitation
JAK: Janus Kinase
LC: Liquid Chromatography
LC-MS: Liquid Chromatography–Mass Spectrometry
LC-MS/MS: Liquid Chromatography–Tandem Mass Spectrometry
LRG: Leucine-Rich Alpha-2 Glycoprotein
MALDI: Matrix-Assisted Laser Desorption/Ionisation
MALDI-TOF: Matrix-Assisted Laser Desorption/Ionisation–Time of Flight
MBC2/FAM62A: Family with Sequence Similarity 62 Member A
MRM: Multiple Reaction Monitoring
MS: Mass Spectrometry
MS/MS: Tandem Mass Spectrometry
OA: Osteoarthritis
PBMC: Peripheral Blood Mononuclear Cell
PMF: Peptide Mass Fingerprinting
Q-TOF: Quadrupole Time of Flight
RA: Rheumatoid Arthritis
RF: Rheumatoid Factor
